# Silvicultural and Ecological Characteristics of *Populus bolleana* Lauche as a Key Introduced Species in the Urban Dendroflora of Industrial Cities

**DOI:** 10.3390/plants14132052

**Published:** 2025-07-04

**Authors:** Vladimir Kornienko, Valeriya Reuckaya, Alyona Shkirenko, Besarion Meskhi, Anastasiya Olshevskaya, Mary Odabashyan, Victoria Shevchenko, Svetlana Teplyakova

**Affiliations:** 1 Scientific Research Laboratory for Monitoring and Forecasting of Donbass Ecosystems, Donetsk State University, 24 Universitetskaya St., 83001 Donetsk, Russia; reutskaya_lerochka@mail.ru (V.R.); alyona.shkirenko@mail.ru (A.S.); 2 Agribusiness Faculty, Don State Technical University, 344000 Rostov-on-Don, Russia; spu-02@donstu.ru (B.M.); olshevskaya.av@gs.donstu.ru (A.O.); modabashyan@donstu.ru (M.O.); vshevchenko@donstu.ru (V.S.); steplyakova@donstu.ru (S.T.)

**Keywords:** poplar, dendroecology, steppe zone, urban environment, stability of trees, biomechanics of woody plants

## Abstract

In this work, we evaluated the silvicultural and ecological parameters of *Populus bolleana* Lauche trees growing in conditions of anthropogenic pollution, using the example of one of the largest megacities of the Donetsk ridge, the city of Donetsk. The objectives of this study included determining the level of anthropogenic load of the territory; conducting dendrological studies to assess morphometric and allometric parameters, age structure, and condition of *P. bolleana* stands under the influence of environmental factors; as well as completing biomechanical studies to assess and predict the mechanical stability of stands. A total of 1109 plants growing in areas with increased anthropogenic load and in the control areas were studied. The model territories of the study were located in the city of Donetsk on Fallen Communards Avenue (length of field routes: 2.6 km) and Ilyicha Avenue (length of field routes: 9.7 km). Control plantings grew on the territory of the Donetsk botanical garden and residential (dormitory) districts of the city. The age structure of *P. bolleana* plantations remained uniform throughout the city for 50–55 years due to the fact that the landscaping was under a single state program. In the steppe zone in the south of the East European Plain, with a high level of anthropogenic load and severe natural climatic factors, the critical age of *P. bolleana* (55 years) was determined. The condition of plantations and their morphometric indices correlate with the level of anthropogenic load of the city (H, Dbase, DBH). Under control conditions, the plants are in good condition with signs of weakening (2 points). Under conditions of increased anthropogenic load, the plants are in a severely weakened condition (3 points). A total of 25% of the plants in the sample are in critical condition (4–5 points). The main damages to the crowns and trunks of plants include core rot, mechanical damage to bark and tissues, the development of core rot through the affected skeletal branch, crown thinning, and drying. *P. bolleana* trees are valued for their crown area and ability to retain dust particles from the air. The analysis of experimentally obtained data on the crown area showed that in the initial phases of ontogenesis, the average deviation in the crown area of plants does not depend on the place of growth. Due to artificial narrowing and sanitary pruning of the crown, as well as skeletal branches dying along the busiest highways, the values do not exceed 22–23 m^2^ on average, with an allometric coefficient of 0.35–0.37. When comparing this coefficient in the control areas, the crown area in areas with a high level of anthropogenic load is 36 ± 11% lower. For trees growing under the conditions of the anthropogenic load of an industrial city and having reached the critical age, mechanical resistance varied depending on the study area and load level. At sites with a high level of pollution of the territory, a significant decrease in indicators was revealed in comparison with the control (mcr—71%, EI—75%, RRB—43%). Having analyzed all the obtained data, we can conclude that, until the age of 50–55 years, *P. bolleana* retains good viability, mechanical resistance, and general allometric ratios, upon which the stability of the whole plant depends. Even with modern approaches and tendencies toward landscaping with exotic introductions, it is necessary to keep *P. bolleana* as the main species in dendrobanocenoses.

## 1. Introduction

A modern industrial city is an “ecological testing ground” in which both natural and anthropogenically transformed ecosystems are present [1,2,3,4,5,6]. Anthropogenic impact leads to the transformation of material and energy flows and changes in ecosystem functions and services [7]. The history of natural environment transformation in the territory of the Donetsk ridge (an upland in the south of the East European Plain) under the intensive influence of human activity on ecosystems is appreciable [8,9,10,11,12,13]. According to the analysis of the spore–pollen complex of plants from the Early Quaternary period, numerous findings of dead remains of woody plants were detected—hornbeam, beech, cypress, etc. The territory of the Donetsk ridge was mostly covered with oak forests with an admixture of hornbeam, elm, maple, and ash (up to 70% according to some sources) at the beginning of the first millennium AD [12]. The high woodiness of the Central Donbas is confirmed by historical archival and cartographic surveys, statistical materials, and scientific surveys. In particular, one of the most original sources, “Map of forests along the Mius River……” (1768), testifies that forests grew in all parts of the hydrographic network of the ridge, which also often extended even beyond the boundaries of the gullies toward the watersheds. At the beginning of the 17th century, with the beginning of settlement of the “wild field” (as indicated in a special map of the western part of Russia by Schubert, 1782, and others), the forest cover of the territory decreased by 20% due to mass felling.

This is confirmed by data on the presence of podzolized black soils and forest soils in places where there are currently no forests, as well as historical names of settlements derived from the names of tree species—Yasinovataya, Olkhovka, Starodubovka, Grabovo, Maple, Dubrovka, Orekhovoye, and beams—Dubovaya, Grabovaya, Biryuchya, Pear, Lipovaya, Aspen, etc., where these species at the present stage of ecosystem development are practically absent. The maximum volumes of destruction and catastrophic reduction of the Donetsk ridge forest cover occurred in the 1790s. The largest forests were allocated for the development of the Black Sea Fleet in the decree of the Senate of 1799. The intensive logging of bayrach and floodplain forests was also associated with the construction and development of the cities of Taganrog, Rostov, and Bakhmut; the construction of mines, factories, and residential buildings; and the plowing of land. Subsequently, the forest destruction rate decreased after the organization of forestry departments (1860s–1870s). At the beginning of the 20th century, the forest cover of the Donetsk ridge averaged only 3%.

The development of industry and economy in the region has led to the emergence of megacities. Windstorms, dust storms, microclimate changes, and environmental pollution have prompted a rethinking of human approaches to interaction with nature. As a consequence, the issue of green building has become acute. Green spaces have become an obligatory element of city beautification and the most significant factor in protecting health and providing recreation for the population. At the state level, a plan was adopted to expand urban forest plantations, artificially create protective strips, including special-purpose areas (e.g., sanitary green zones between industrial areas), linear plantings along highways, as well as the transformation of a number of urban areas into parks and public gardens. The landscaping of industrial cities of the Soviet Union was carried out according to a unified program, as a result of which there were peculiarities in the formation of the dendroflora of industrial cities, including in the south of the East European Plain.

*Populus* L. species have been studied and used everywhere in green building since the 1950s, and not only within the USSR. The problem of the sustainability of *Populus* L. species in the conditions of industrial cities has a world-wide scope: the accumulation of heavy metals by plants and the phytoremediation of soils using poplars (the best term is poplars) [14,15,16,17,18,19,20,21,22,23], adaptation to stress among poplars at molecular levels [24,25,26,27,28,29,30], ecosystem services of poplars [31,32], and others. This genus of woody plants, *Populus bolleana* Lauche, has performed well in cities with a high level of anthropogenic load in terms of ecological functions. The plant is native to Central Asia, highly ornamental, frost- and winter-hardy, drought-resistant (which is especially valuable in the steppe zone), shade-tolerant, fast-growing, and retains a large volume of dust. It is considered to have a life expectancy of up to 70 years in metropolitan conditions, which has received separate scientific interest and provides new opportunities for research. Some species of the genus *Populus* L. are also worth mentioning, which have made a significant contribution to the formation of the green framework and appearance of industrial cities of the Donetsk ridge—*Populus nigra* L. and *Populus nigra* var. *italica* (Moench) Koehne [33,34]. Scientifically substantiated approaches to the use of fast-growing species introduced in landscaping have helped achieve positive dynamics in the territory’s forest cover, increasing from an average of 2.8% in 1946 to 5.8% in 1976 and 7.2% in 2010.

At the present stage of the development of industrial cities, a number of problems related to the stability and development of biological systems have appeared [35,36,37,38,39,40,41,42,43,44,45,46,47,48,49]. Especially acute is the issue of the integrity of dendrocenoses, including fast-growing species of the genus *Populus* L.

In general, ecosystem stability is considered as a ratio between the magnitude of a stressful impact and the degree of resulting damage to its various components, such as organisms, populations, biocoenoses, etc. It also depends on the ability of organisms to maintain relative constancy of the internal environment—homeostasis within a certain range of external impacts. Taking into account the fact that plants were planted en masse more than 50 years ago and grew under the constant influence of anthropogenic factors, there are threats to the loss not only of single specimens but of cascade processes. For the transformed ecosystems of industrial cities, studies to assess the state of species, determine the age threshold, study adaptation mechanisms, and ecological and biological features of growth under the influence of unfavorable environmental factors have become increasingly important in recent years.

Due to the fact that artificial afforestation is still not sufficient, measures aimed at the compensation of the forest cover loss of the Donetsk ridge and the creation of urban plantations are often neglected. There is a low level of naturalization and, quite naturally, their parameters are far from those of indigenous forest types. The aim of the present study was to assess the silvicultural and ecological parameters of *P. bolleana* trees growing under anthropogenic pollution conditions, using the example of one of the largest megacities on the Donetsk ridge, the city of Donetsk.

The objectives of this study were to determine the level of anthropogenic load of the territory using one of the most important sources of physical and chemical pollution of the environment—motor vehicles and noise pollution; to conduct dendrological studies to assess the morpho-metric and allometric parameters, age structure, and condition of *P. bolleana* Lauche stands under the influence of environmental factors; and to conduct biomechanical studies to assess and predict the mechanical stability of stands.

## 2. Results

### 2.1. Analysis of Plant Growth Conditions

**Figure 1:**
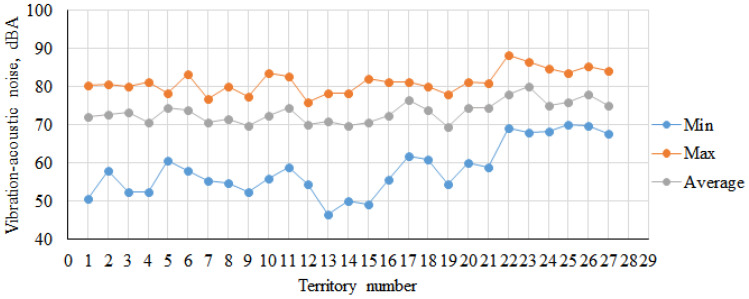
Vibration–acoustic noise pollution in Donetsk city along Fallen Communards Avenue (No. 1–21) and Ilyicha Avenue (No. 22–27).

**Figure 2:**
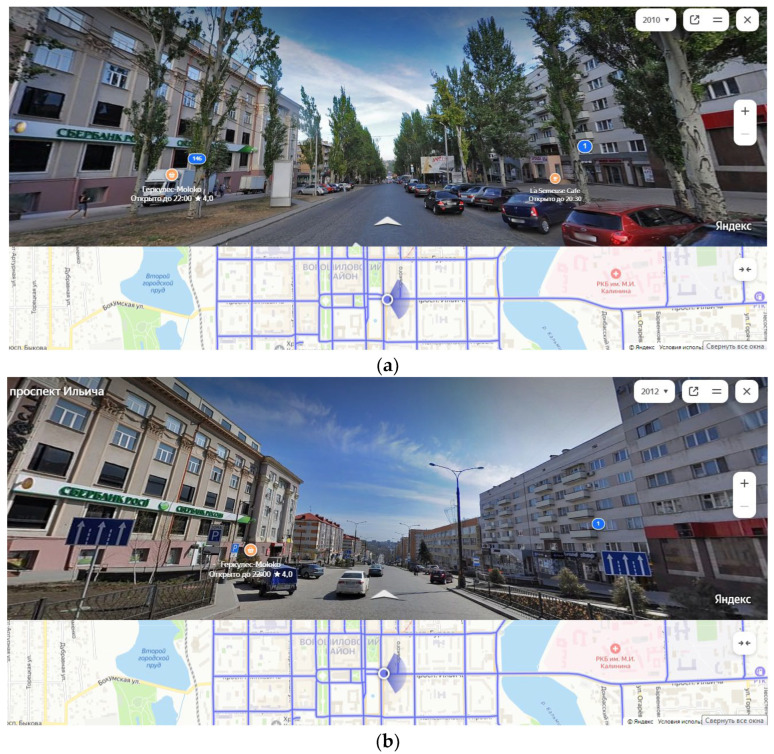
Changes in the structure of the biotic components of the urban ecosystem along Ilyicha Avenue due to construction activities. Notes: (**a**) 2010; (**b**) 2012.

The results of the anthropogenic load assessment of the studied sections are presented in Table 1 and Figure 1 and Figure 2. The traffic flow intensity reflects a high load level on green spaces growing along highways. The distribution of vehicle types in the overall urban traffic is typical of metropolises: a significant share of foreign-manufactured passenger vehicles with reduced noise levels (65%) and domestically produced passenger vehicles (~20%). Vehicles causing critical noise pollution (~90 dBA) account for 13% of the total.

The analysis of noise regulation compliance in sections A (No. 1–10) and B (No. 21–11) revealed significant exceedances in both equivalent and maximum noise levels (Table 2). On average, equivalent noise in sections A and B exceeded permissible limits by 11.5 ± 3.2%, while maximum noise exceeded by 14.7 ± 2.4%. Variations in noise pollution depended on the following factors: the road inclination angle (a steep downhill slope from No. 1 to 10), the presence of high-profile speed bumps, road surface defects (No. 20–21 include a tram rail depot with degraded rail infrastructure), and vegetation removal. At No. 11–12, poplars and other tree species were cleared along an extended road segment due to post-2010 construction. In section A, No. 10, over half of the *P. bolleana* plants were lost due to weakened vitality and adverse 2023 climatic events, further degrading ecological noise mitigation. By 2025, construction work to replace curbstones had commenced, with observed root system damage, skeletal branch injuries, and mechanical trunk damage observed in many 50–60-year-old trees.

The analysis of equivalent noise levels identified critical exceedances in sections C (No. 22–24) and D (No. 25–27) at +28.5 ± 1.8% (Figure 1). Peak sound pollution was in the range of +15–20%, with average exceedances of +17.6 ± 1.7%. Noise variability was influenced by a steep downhill slope from Lenin Square to the Kalmius River (one-way traffic) and cobblestone pavement (limiting vehicle speed but amplifying vibration–acoustic noise at No. 22 and 27).

Cobblestone paving work was conducted in 1945 and during road reconstruction in 2011. Following the repairs, vibration–acoustic noise pollution increased in the area. Additionally, a decision to remove all *P. bolleana* trees (Figure 2) was made by the city authorities. After, the local microclimate shifted (e.g., increased average air temperature, decreased humidity, etc.).

Kalmius River along Ilyich Avenue is crossed by Victory Bridge, which lacks green zones and technical noise/vibration-damping measures, contributing to the propagation of elastic waves. Since 2024, construction activities on the studied site have included road expansion, asphalt laying, pedestrian path construction, and curbstone replacement. Some *P. bolleana* plants were removed, while most sustained mechanical damage from construction, as described in the earlier case.

**Figure 3:**
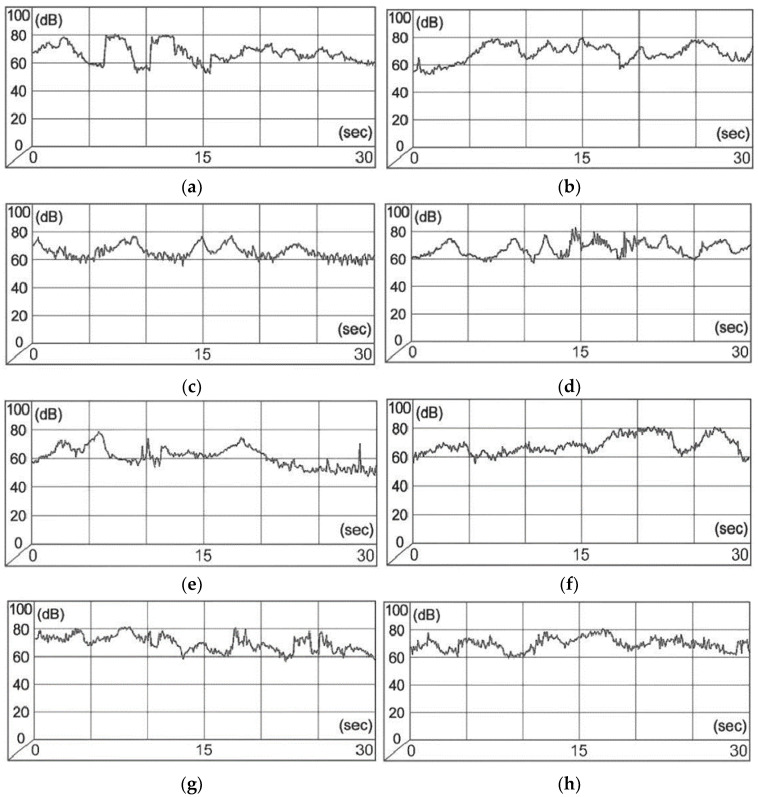
Time-resolved amplitude–frequency spectra of various vehicle types in study areas along Fallen Communards Avenue. Notes: (**a**) section A (No. 1); (**b**) section A (No. 3); (**c**) section A (No. 7); (**d**) section A (No. 10); (**e**) section B (No. 19); (**f**) section B (No. 16); (**g**) section B (No. 11); (**h**) section B (No. 20).

Figure 3 displays typical 30 s time-resolved amplitude–frequency spectra of various vehicle types in study sections A and B. Beyond elevated noise levels (Fallen Communards Avenue: Ieq. = 72.2 ± 2.1 dBA, Imax = 80.0 ± 2.1 dBA; Ilyicha Avenue: Ieq. = 77.0 ± 2.0 dBA, Imax= 85.0 ± 1.8 dBA), the amplitude component of traffic flow is critical. For biological organisms, minimum and peak values of anthropogenic or natural–climatic factors, as well as the frequency of such cycles impacting biosystems, are particularly consequential.

### 2.2. Dendrological Research

**Figure 4:**
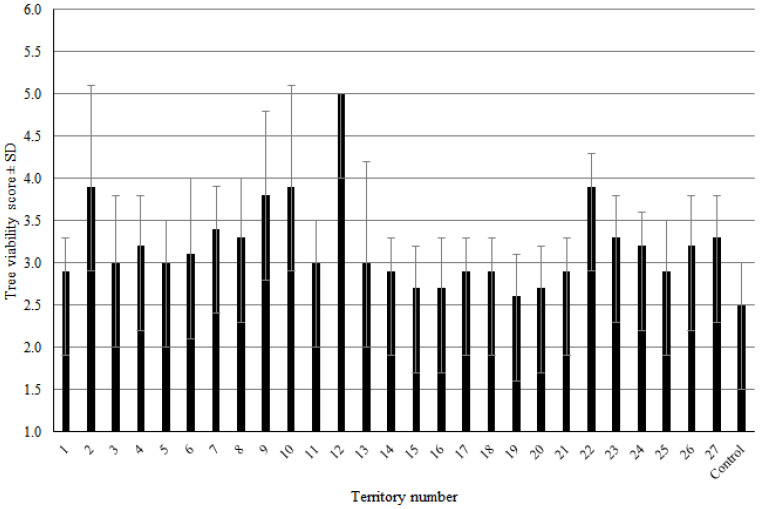
Tree viability in Donetsk city along Fallen Communards Avenue (No. 1–21) and Ilyicha Avenue (No. 22–27).

Based on field studies and subsequent office data processing, the average dendrometric parameters of *P. bolleana* were summarized for each study section (Figure 4, Table 2). The trunk diameter at the base and at 1.3 m height differed significantly (*p* < 0.05) by an average of +25% in sections A and B compared to C and D, while the tree height was 1 m shorter. This survival strategy may relate to mechanical stability in linear plantings along highways. In control zones, the tree height was ~10% greater, but the trunk diameter showed no significant difference from sections A and B.

**Figure 5:**
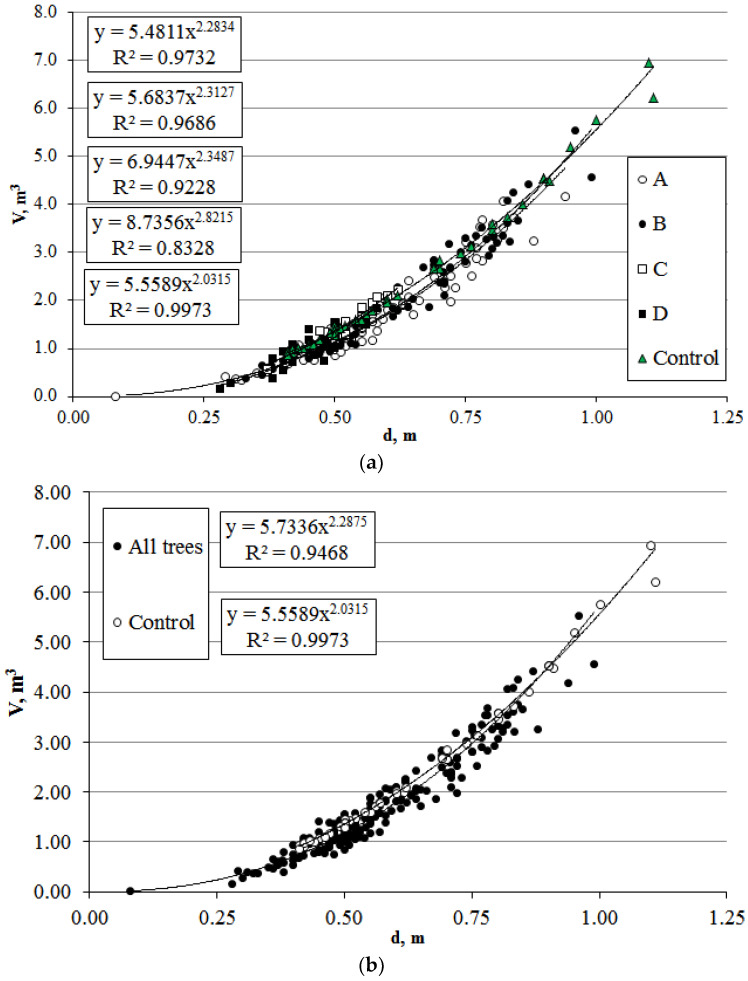
Relationship between trunk volume (V) and diameter for *P. bolleana* growing in control and urban areas (sections A–D). Comment: (**a**) different study areas; (**b**) general dependence for all experimental areas and control group.

**Figure 6:**
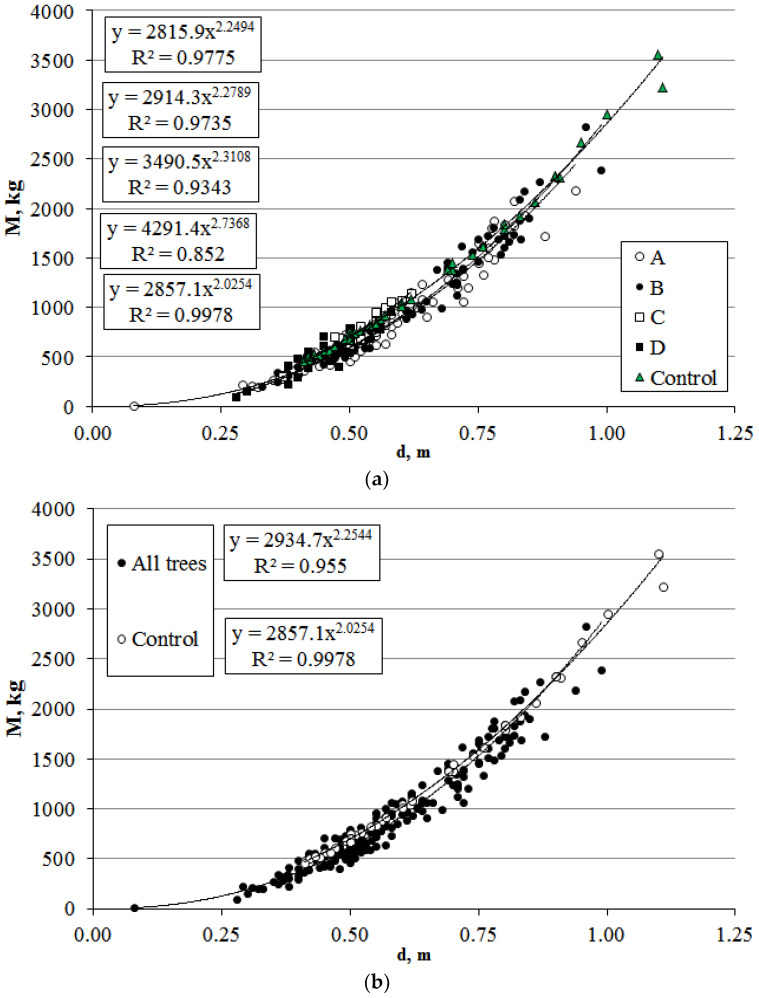
Relationship between total aboveground phytomass and diameter for *P. bolleana* growing in control and urban areas (sections A–D). Comment: (**a**) different study areas; (**b**) general dependence for all experimental areas and control group.

Allometric relationships between stem volume and the aboveground phytomass of *P. bolleana* in control (Control) and experimental sections (sections A–D) were analyzed. The stem volume was significantly lower in trees under anthropogenic loads: 27% (section A), 8% (section B), 38% (section C), and 54% (section D). The regression followed a power-law model with high determination coefficients (R^2^ = 0.83–0.99) (Figure 5). The simulated aboveground phytomass correlated with stem volume (V), showing differences from the control of 26% (A), 7% (B), 38% (C), and 53% (D) (Figure 6). Despite mean value disparities, allometric trends (power-law regression) for stem volume and phytomass relative to plant size were consistent across sections. The allometric diameter-to-height ratio (d/l) was high (0.04–0.05) for both control and experimental groups.

The age structure of *P. bolleana* stands is uniform citywide, including model sections, as industrial cities in the USSR were greened under a unified program that prioritized *Populus* L. species (mainly *P. bolleana*) from the 1950s onward. Since greening followed standardized protocols, Donetsk city serves as a representative model, and these data are extrapolatable to other industrial cities in the steppe zone of the southern East European Plain.

**Figure 7:**
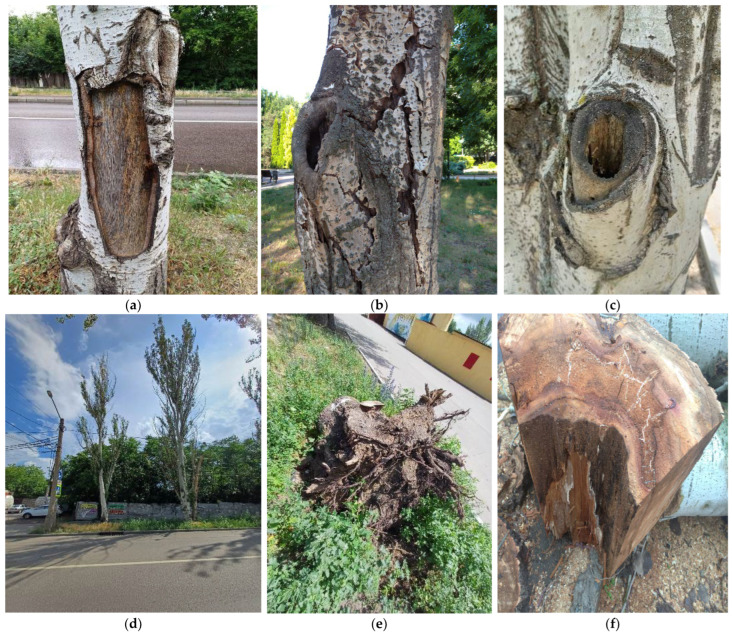
Typical damage types in *P. bolleana* after reaching critical age. Notes: (**a**) Mechanical trunk damage and large open wounds facilitating pest entry; (**b**) natural bark detachment from the trunk; (**c**) heart rot development through infected skeletal branches; (**d**) crown thinning, trunk cracks, and dead tops; (**e**) tree uprooting with root plate after a February 2024 snowstorm; (**f**) hidden heart rot (cross-section, April 2025).

**Figure 8:**
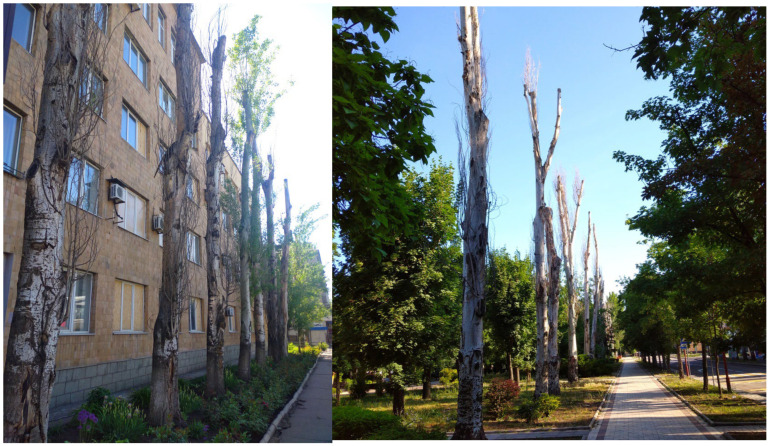
Example of dead *P. bolleana* trees following severe crown reduction in weakened specimens aged 50–55 years (biological effect typical for all linear plantings in industrial cities).

Several reasons explain the use of this species for urban greening: (a) hardiness: trees exhibit high adaptability, tolerate diverse soil compositions, and require minimal maintenance; (b) they have a rapid growth rate; (c) air filtration: poplars effectively capture airborne dust particles in large cities and industrial centers; (d) space efficiency: the species’ elongated, compact form allows dense planting, reducing open spaces that propagate noise, vibrations, and toxin penetration into residential areas. The poplar’s lifespan was assumed to average ~100 years with sustained vitality. However, accelerated aging under high anthropogenic loads and harsh natural-climatic factors of the Donbas region reduces the critical age of *P. bolleana* in industrial cities to 55 years (maximum 60 years). Plants cannot reach their natural ecosystem lifespan. In linear plantings, viability declines sharply after 50 years (Figure 7). The dominant age group averaged a viability score of 3.2 ± 0.2 points (severely weakened state). Retaining such trees in industrial urban stands increases the risks of uprooting and branch breakage, particularly during snow/ice storms (Figure 6 D). Recommendation: proactive replanting should begin when *P. bolleana* reaches 45–50 years, as extreme crown reduction or sanitary pruning fails to extend lifespan or fully restore ecological functions (Figure 8).

The analysis of crown condition in study areas revealed a significant incidence (≥70%) in external damage (accounting for >10% of the total crown volume) in high anthropogenic load zones (sections B and D) and select areas of section A (No. 4, 6, 8–10) and section B (No. 12, 13, 17, 19–21).

**Figure 9:**
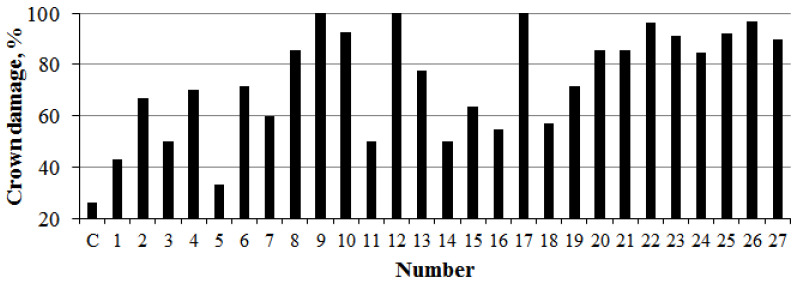
Proportion of trees (%) with at least one of the listed crown damages: crown thinning, trunk cracks, and dead tops.

**Figure 10:**
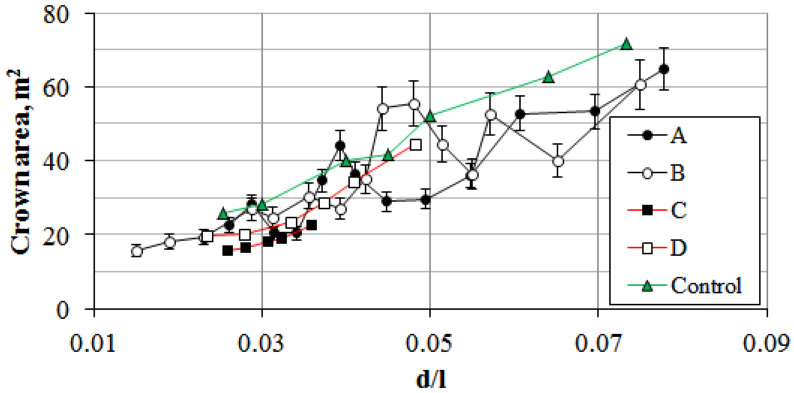
Relationship between crown area of *P. bolleana* and the allometric coefficient of height-to-trunk diameter ratio (d/l) in Donetsk city.

*P. bolleana* trees are valued for their crown area and ability to capture airborne dust particles. The analysis of experimentally derived crown area data revealed that during early ontogenetic stages, crown area deviations are location-independent. However, due to artificial crown narrowing and sanitary pruning (caused by skeletal branch dieback along heavily trafficked highways; Figure 9), crown areas in sections C and D averaged 22–23 m^2^, with an allometric coefficient of 0.35–0.37. Compared to control zones, the crown area in sections A and B was 19 ± 7% lower for this coefficient and 36 ± 11% lower in sections B and C (Figure 10).

**Figure 11:**
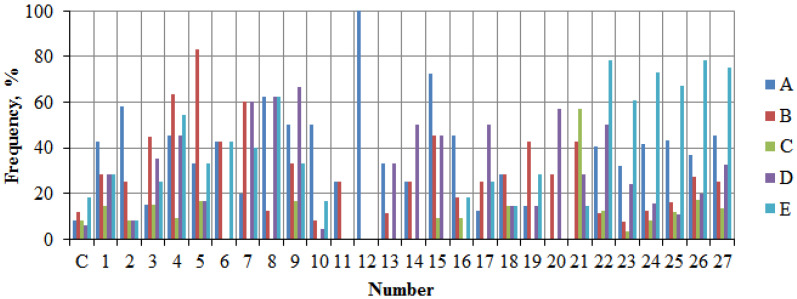
Incidence of stem defects in *P. bolleana*. Key: A—stem rot; B—canker/growth; C—hollow; D—mechanical bark damage and bark detachment; E—frost cracks.

The analysis of trunk damage in *P. bolleana* trees identified key defects and compared their prevalence across study areas. The most critical defects impacting tree viability and hazard potential were stem rot (Figure 11, bar A) and hollows (often entry points for xylophagous insects such as *Sesia apiformis* Cl., *Agrilus virilis* L., *Pulvinaria betulae* L., *Saperda carcharias* L., *Cossus cossus* L., and *Tremex fuscicornis* F.) (Figure 11, bar C), along with mechanical bark injuries (Figure 11, bar D).

In areas with high anthropogenic loads, 40% of the stand (50–55-year-old trees) exhibited stem rot (5-fold higher than control), 25% showed mechanical trunk damage (4-fold higher than control), and 9% had primary skeletal branch damage colonized by xylophagous insects typical of the southern East European Plain, leading to hollow formation.

### 2.3. Biomechanical Research

**Figure 12:**
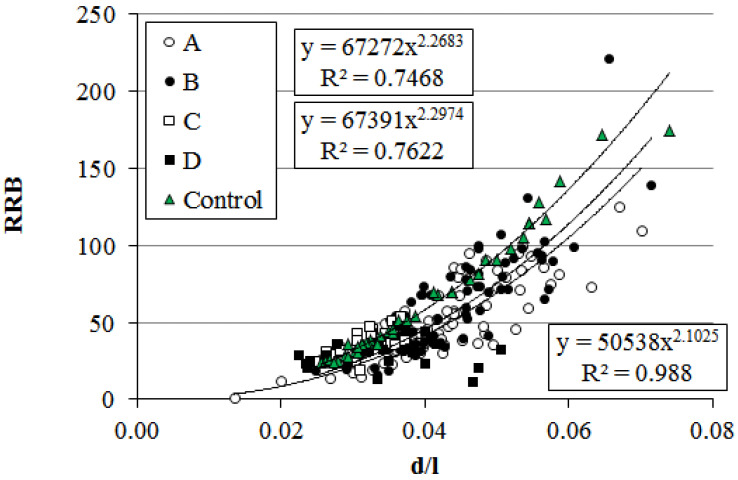
Relationship between relative bending resistance and stem diameter-to-length ratio in *P. bolleana* growing in control (Control) and experimental (A, B, C, D) Sites. Comment: Y-axis (RRB) data are given in “number·10^3^” format.

The relative resistance to bending (RRB) depends directly on the modulus of elasticity. Since RRB increases quadratically with trunk thickness (Figure 12), dynamic and static loads primarily affect trees with minimal trunk diameter-to-height ratios and the lowest structural resilience. For *P. bolleana* trees in sections C and D under elevated anthropogenic loads, RRB values were lowest, differing from controls by 43% on average. The relationship followed a power-law model for all trees, with control stands exhibiting a high determination coefficient (R^2^ = 0.99). The values of the experimental groups were generally located below the curve.

RRB reflects changes in tree stability under mechanical stress but does not quantify critical mass or size thresholds for irreversible trunk bending or breakage. To address this, calculated parameters like flexural stiffness (EI), ultimate permissible load (Pcr), and critical mass (mcr) can be applied. Like RRB, these mechanical stability metrics depend on the physico-mechanical properties of living plant tissues.

**Figure 13:**
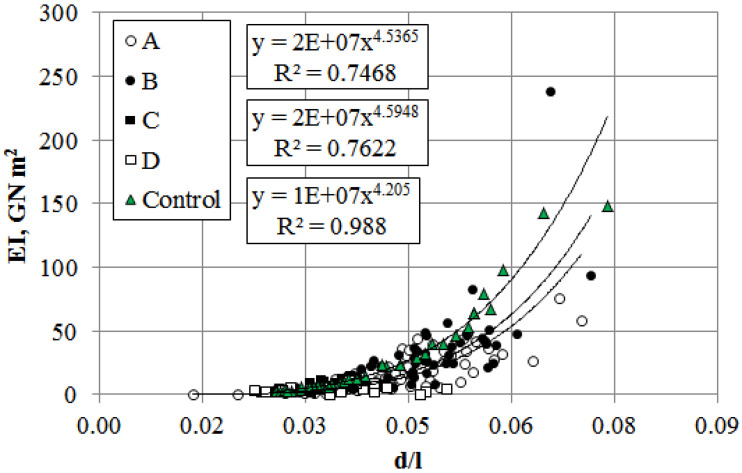
Relationship between flexural stiffness and diameter-to-stem-length ratio of *P. bolleana* growing in control (Control) and experimental (A, B, C, D) Sites. Comment: Y-axis (EI) data are given in “Number·10^6^” format.

Trees most at risk of breakage under mechanical loads are those stressed by elevated anthropogenic loads and light competition (Figure 13). Their hazard potential increases during precipitation and ice accumulation. Under dynamic factors, the critical load further decreases by ~20% [50].

For trees growing under anthropogenic loads, the flexural stiffness (EI) varied across study sites and load intensity. Significant reductions in mechanical stability compared to controls were identified in highly polluted sections D and C (79% and 72%, respectively), with section A showing a 40% decrease. Section B exhibited a minor 9% EI reduction, while other parameters (Pcr, mcr, and RRB) showed no significant deviation from controls.

**Figure 14:**
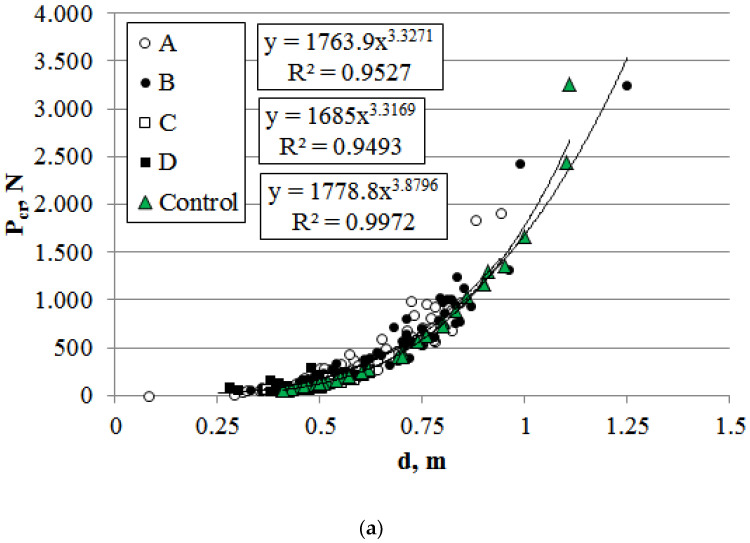

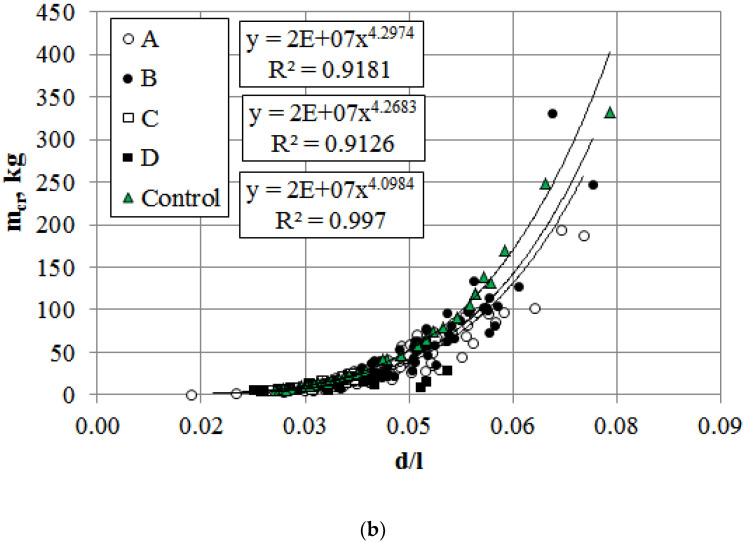
Relationship between ultimate permissible load (**a**) and trunk diameter and critical mass (**b**) and diameter-to-stem-length ratio for *P. bolleana* growing in control (Control) and experimental (A, B, C, D) sites. Comment: Y-axis (m_cr_, P_cr_) data are given in “Number·10^3^” format.

The critical mass and load capacity under ecological stressors were 70% lower for sections C and D, and 20% lower for section A (Figure 14a,b). Power-law dependencies differed in slope angles: trees in control areas exhibited steeper increases in critical mass with rising allometric d/l ratios. The smallest critical mass increase relative to d/l ratios was observed in sections C and D.

## 3. Materials and Methods

### 3.1. Study Subject

Study subject—The Bolle poplar (*Populus bolleana* or *Populus alba* L. var. *bolleana* Lauche), a variety of white poplar. This species is highly ornamental, distinguished by its silver foliage, smooth white (graying) trunk, and pyramidal crown. It produces substantial foliage and plays a key role in shaping a favorable urban microclimate for residents. Growth rate: fast. Origin: Central Asia. Biogeography: Europe, North Africa, West Asia. In Donetsk city, it is one of the most prevalent tree species, constituting ~17% of the urban tree population. *P. bolleana* exhibits strong physico-mechanical wood properties, including resistance to splitting, standard density, and compressive strength along the grain.

### 3.2. Study Area

**Figure 15:**
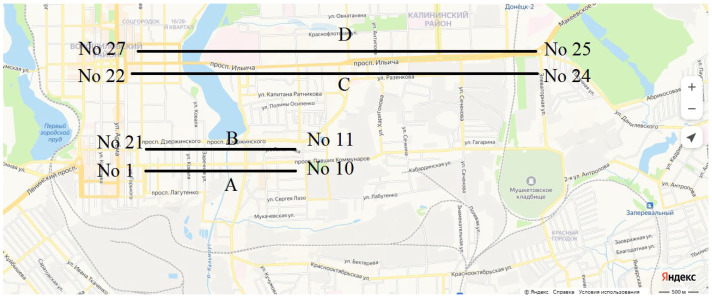
Study area along the central highways of the industrial city of Donetsk.

Model study areas in Donetsk city include Fallen Communards Avenue (2.6 km) and Ilyicha Avenue (9.7 km). Four study sections were delineated during field surveys (Figure 15):

- Fallen Communards Avenue: section A (No. 1–10) and section B (No. 11–21);
- Ilyicha Avenue: section C (No. 22–24) and section D (No. 25–27).

Control stands were located in the Donetsk Botanical Garden and residential (low-traffic) city districts. A total of 1109 plants were assessed in anthropogenically polluted areas.

### 3.3. Analysis of Anthropogenic Load on the Study Area

Vehicle traffic intensity along the study sections was assessed by counting specific vehicle types passing measurement points per unit of time.

Noise levels were measured using a portable Benetech noise meter (±1 dBA accuracy). Measurements were conducted during peak traffic hours on weekdays per GOST* 23337-2014 at 27 points (No. 1–27). The following regulatory documents were applied:

- GOST (Russian State Standard) 23337-2014: Noise. Methods for Measuring Noise in Residential Areas and in Premises of Residential/Public Buildings;
- SanPiN (Sanitary Regulations and Standards) 1.2.3685-21: Hygienic Standards and Requirements for Ensuring Human Safety Against Environmental Factors;
- SP 51.13330.2011: Noise Protection (Updated SNiP 23-03-2003), according to which the equivalent sound level during the daytime should not exceed 55 dBA and the maximum sound level should not exceed 70 dBA in areas directly adjacent to residential buildings. For the equivalent value when measuring noise 2 m from the highway, it is permissible to accept 10 dBA higher (correction = +10 dBA).

### 3.4. Dendrological Research Methods

Viability assessment was conducted using the integrated Alekseev scale with modifications for trunk and crown damage [51]: 1 point—healthy plant; 2—weakened; 3—severely weakened; 4—dying; 5—deadwood.

Visual inspection data for studied trees were documented using a Nikon Coolpix S2600 camera (Japan, 2009). Subsequent office processing and image analysis were performed in AxioVision Rel. 4.8 software with reference scaling. Over 1000 digital images were analyzed to study crown architecture and stem defects. Trunk diameter was measured with a Haglöf Mantax caliper (Sweden, 2024).

Phytomass assessment and modeling of *P. bolleana* tree fractions were based on Demakov’s method [52].

The aboveground phytomass (M, kg) of foliated *P. bolleana* trees was calculated using Equation (1):

M = a × 10^−2^ × h^b^ × (d + 1)^2^, (1)

Stem volume (V, m^3^) was determined via Equation (2):

V = a × 10^−5^ × h^b^ × (d + 1)^2^, (2)

where a, b—model-specific constants derived from regression analysis; h—tree height (m); d—trunk diameter at breast height (1.3 m; cm).

### 3.5. Biomechanical Research Methods

Parameters of mechanical stability (EI, RRB, and P_cr_ и m_cr_) were calculated using the following formulas:

1. Resistance to bending under dynamic/static loads [53,54]:
  Bending resistance = E × I,(3)
2. Relative resistance to bending (RRB) [54]:
  RRB = r^2^ × E/4 × ρ, (4)
  where E—modulus of elasticity of living wood tissues (N·m^2^), I—second moment of area (π × r^4^/4); ρ—density of living tissues (kg/m^3^); r— tree trunk radius (m).
3. Critical mass (m_cr_) and ultimate permissible load (P_cr_)—parameters that reflect specific values of mass (kg or N), under the action of which the trunk of a tree plant or its skeletal branches begin to deform or break off under the action of wind or gravitational loads [54]:
  P_cr_ = π^2^ EI/(2l^2^), (5)
  m_cr_ = P_cr_/g. (6)
  where EI—bending resistance, l—length (m), and g—gravitational acceleration (9.81 m/s^2^).

The Microsoft® Excel® LTSC MSO (version 2505, Assembling 16.0.18827.20102) (Microsoft Corporation) was used for statistical data processing. The dependence of the critical mass (mcr), bending resistance (EI), and RRB on the d/l coefficient and trunk diameter for the studied plants was established using a power regression model.

## 4. Conclusions

Due to accelerated aging under high anthropogenic loads and harsh natural–climatic factors of the steppe zone in the southern East European Plain, the critical age of *P. bolleana* in industrial cities is 55 years, with a maximum individual lifespan of 60 years. Viability declines sharply after trees reach 50 years, averaging 3 points (severely weakened state). Dying or critically compromised trees accounted for 25 ± 7% of stands in zones with high anthropogenic loads.

The trunk diameter was significantly larger (*p* < 0.05) by 25% in sections A and B compared to C and D, while the tree height was 1 m shorter. This survival strategy enhances mechanical stability in linear plantings along highways. In control zones, the tree height was ~10% greater, but the trunk diameter showed no significant difference from sections A and B.

The stem volume and total aboveground phytomass were significantly lower in trees under anthropogenic loads: 26% (section A), 7% (section B), 38% (section C), and 53% (section D). Despite mean value disparities, allometric trends (power-law regression) for the stem volume and phytomass relative to plant size were consistent across zones. The allometric diameter-to-height ratio (d/l) was high (0.04–0.05) for both control and experimental groups.

Crown condition analysis revealed significant external damage incidence (≥70%) in high-load areas. Additionally, 40% of 50–55-year-old stands in these zones exhibited stem rot, 25% had mechanical trunk damage, and 9% displayed primary skeletal branch damage colonized by xylophagous insects typical of the southern East European Plain steppe zone, leading to hollow formation.

For *P. bolleana* trees under anthropogenic loads at a critical age, mechanical stability varied by study site and load intensity. Highly polluted areas showed drastic reductions versus controls (mcr—71%, EI—75%, and RRB—43%). Comprehensive analysis confirms that *P. bolleana* retains high viability, mechanical stability, and critical allometric ratios (essential for overall resilience) until ages 50–55. Despite modern trends favoring exotic introduced species, *P. bolleana* (male specimens) must remain dominant in industrial urban dendrocenoses.

## Figures and Tables

**Table 1:** Average traffic flow intensity on weekdays along Fallen Communards Avenue (A) and Ilyicha Avenue (B) in Donetsk.

Territory	Mode of Transport, Average Value ± SD unit/hour						Total, unit/hour Average Value ± SD
	Cargo Truck		Passenger				
	Light	Heavy	Passenger vehicles			Buses	
			Foreign-Manufactured Passenger Vehicles	Domestically Produced Passenger Vehicles	SUV		
A	34 ± 3	16 ± 2	766 ± 62	294 ± 29	94 ± 13	17 ± 3	1221 ± 115
B	77 ± 5	17 ± 9	968 ± 67	198 ± 13	102 ± 7	63 ± 5	1424 ± 60

**Table 2:** Silvicultural and taxational characteristics of *P. bolleana* in Donetsk city.

Number	Height ± SD, m	D_base_ ± SD, m	DBH ± SD, m	Age ± SD, Year
Section A				
1	13.6 ± 1.7 *	0.58 ± 0.15	0.50 ± 0.11	52.0 ± 3.7
2	14.0 ± 2.6 *	0.57 ± 0.13	0.52 ± 0.15	51.7 ± 4.0
3	15.5 ± 1.7	0.59 ± 0.21	0.58 ± 0.20	49.3 ± 10.2
4	13.5 ± 1.3 **	0.49 ± 0.11 *	0.45 ± 0.12 *	48.7 ± 4.4 *
5	12.6 ± 1.3 **	0.47 ± 0.08 *	0.43 ± 0.06 *	46.5 ± 4.7 *
6	14.0 ± 1.8 *	0.61 ± 0.11	0.55 ± 0.10	51.3 ± 3.5
7	13.8 ± 0.6 *	0.59 ± 0.11	0.53 ± 0.07	52.4 ± 4.3
8	13.6 ± 1.1 *	0.59 ± 0.09	0.54 ± 0.10	49.6 ± 3.3
9	12.0 ± 0.7 **	0.55 ± 0.17	0.46 ± 0.09 *	45.8 ± 4.9 *
10	12.2 ± 2.5 **	0.61 ± 0.16	0.58 ± 0.12	53.4 ± 3.5
Section B				
11	13.8 ± 2.9 *	0.60 ± 0.19	0.34 ± 0.07 *	50.0 ± 7.0
12	–	0.62 ± 0.09	–	53.9 ± 2.2
13	14.7 ± 2.7 *	0.69 ± 0.27	0.63 ± 0.17	51.1 ± 5.5
14	13.8 ± 1.7 *	0.60 ± 0.09	0.55 ± 0.07	50.9 ± 3.2
15	13.0 ± 0.9 *	0.65 ± 0.25	0.57 ± 0.23	50.6 ± 4.2
16	14.3 ± 1.3 *	0.54 ± 0.13	0.51 ± 0.15	50.9 ± 3.8
17	16.9 ± 0.8	0.70 ± 0.08	0.65 ± 0.09	52.5 ± 2.7
18	15.3 ± 1.5	0.75 ± 0.06	0.70 ± 0.09	55.0 ± 2.9
19	15.2 ± 1.2	0.74 ± 0.14	0.69 ± 0.10	52.1 ± 3.9
20	14.2 ± 2.7	0.60 ± 0.23	0.52 ± 0.19	49.3 ± 5.3
21	12.8 ± 1.9 **	0.51 ± 0.12 *	0.48 ± 0.14 *	47.9 ± 4.9 *
Section C				
22	15.1 ± 1.6	0.43 ± 0.043 **	0.38 ± 0.048 **	48.7 ± 11.7
23	16.1 ± 1.3	0.53 ± 0.033 *	0.48 ± 0.032 *	59.5 ± 3.3
24	16.5 ± 1.4	0.54 ± 0.057 *	0.48 ± 0.057 *	56.3 ± 3.2
Section D				
25	13.2 ± 3.2 **	0.45 ± 0.053 **	0.40 ± 0.050 **	42.1 ± 7.8 **
26	13.4 ± 3.1 **	0.49 ± 0.099 **	0.44 ± 0.090 **	49.7 ± 10.7
27	16.3 ± 2.0	0.46 ± 0.029 **	0.41 ± 0.03 **	59.3 ± 4.8
Control plantings				
**-**	16 ± 0.5	0.60 ± 0.09	0.52 ± 0.14	53 ± 2.5

Comment: *—reliable difference between sample and control, *p* < 0.05; **—*p* < 0.01.

## Data Availability

The original contributions presented in the study are included in the article; further inquiries can be directed to the corresponding author.

## Abbreviations

EI	Flexural stiffness.
Pcr	Ultimate permissible load.
mcr	Critical mass.
RRB	Relative resistance to bending.
